# Self-oxygenation mesoporous MnO_2_ nanoparticles with ultra-high drug loading capacity for targeted arteriosclerosis therapy

**DOI:** 10.1186/s12951-022-01296-x

**Published:** 2022-02-19

**Authors:** Weidong Sun, Yiyan Xu, Ye Yao, Jie Yue, Zhen Wu, Haocheng Li, Guanghui Shen, Yan Liao, Haiyang Wang, Wenhu Zhou

**Affiliations:** 1 Department of Vascular and Interventional Surgery, The First Affiliated Hospital of Harbin Medical University, Harbin, 150001 Heilongjiang China; 2 Department of Vascular Surgery, The First Affiliated Hospital of Guangzhou Medical University, Guangzhou, 510120 Guangdong China; 3 Xiangya School of Pharmaceutical Sciences, Central South University, Changsha, 410013 Hunan China

## Abstract

Atherosclerosis (AS) is a leading cause of vascular diseases that severely threats the human health due to the lack of efficient therapeutic methods. During the development and progress of AS, macrophages play critical roles, which are polarized into pro-inflammatory M1 phenotype to excrete abundant cytokines and overproduce reactive oxygen species (ROS), and take up excess amount of lipid to form foam cells. In this work, we developed a MnO_2_-based nanomedicine to re-educate macrophages for targeting AS therapy. The MnO_2_ was one-pot synthesized under mild condition, showing intrinsic catalase-mimic activity for self-oxygenation by using endogenous H_2_O_2_ as substrate. Moreover, the mesoporous structure as well as the abundant metal coordination sites in MnO_2_ structure facilitated the loading of an anti-AS drug of curcumin (Cur), achieving extraordinarily high drug loading capacity of 54%. Cur displayed a broad spectrum of anti-oxidant and anti-inflammatory capabilities to repolarize M1 macrophages into M2 phenotype, and the catalytic MnO_2_ recovered the function of lipid efflux transporter to remove lipid from cells by suppressing HIF-1α. Collectively, the nanocarrier and the payload drug functioned as an all-active nanoplatform to synergistically alleviate the syndromes of AS. In ApoE−/− mice model, the nanosystem could significantly prolong the circulation half-life of Cur by sixfold, and enhance drug accumulation in atherosclerotic lesion by 3.5-fold after intravenous injection by virtue of surface hyaluronic acid (HA) modification. As a result, a robust anti-AS efficacy was achieved as evidenced by the decrease of atherosclerotic lesion, plaque area, lipid level.

**Graphical Abstract:**

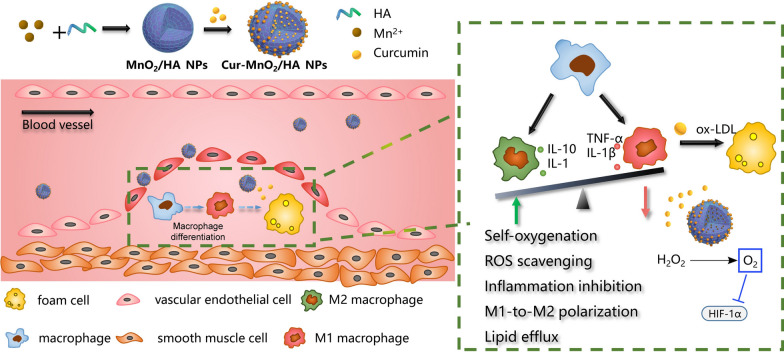

**Supplementary Information:**

The online version contains supplementary material available at 10.1186/s12951-022-01296-x.

## Introduction

Arteriosclerosis (AS), a chronic and progressive vascular disease, is the major contributor of lethal cardiovascular diseases. AS is featured by dysfunction of lipoprotein cholesterol metabolism and the accumulation of plaques underneath the inner wall of the vessel, which is composed of lipids and inflammatory cells [1]. It is generally accepted that the excessive intake and accumulation of lipid (mainly low-density lipoprotein, LDL) is the initial step of AS, which is internalized by macrophages to trigger a series of subsequent pathological processes, such as chronic inflammatory response, artery intima injury, foam cells formation, and finally, the development of plaques [2]. The early and steady plaques could exist for many years, while the major risk is the advance and vulnerable plaques that rupture to cause ischemic complications, such as sudden cardiac death, stroke, acute myocardial infraction, and other severe outcomes. Therefore, intervention the plaques of AS at early stages is critically important to control the disease progress and save the patients’ lives.

During the progress of AS, macrophages are the central cells in the development, progression, and stability of plaques [3]. The monocytes are recruited into AS site and differentiate into classical pro-inflammatory M1 macrophages, which create a pro-atherogenic local via different mechanisms, such as cytokines excretion and reactive oxygen species (ROS) generation. Meanwhile, the macrophages take up excess amount of oxidized low-density lipoprotein (ox-LDL) to form foam cells [4]. Normally, the internalized lipid can be digested and then removed from macrophages by different transporters, such as ATP-binding cassette transporter A1 (ABCA1), and G1 (ABCG1) [5]. However, AS region is pathologically featured with extreme hypoxia, which cause the up-regulation of hypoxia-inducible factor 1α (HIF-1α). HIF-1α could directly participate the progression of AS via promoting inflammation and the proliferation of smooth muscle cell (SMC) and endothelial cell (EC) [6], and importantly, decrease the cholesterol efflux via inhibiting cholesterol esterification and the function of ABCA1 [7, 8]. As such, the balance of lipid influx and efflux is broken, and the dysregulation of lipid metabolism in turn facilitates the M1 macrophages transformation, as well as compromises their critical immune functions. The lipid overloaded macrophages tend to transform into foam cells with diminished migration capability, and large amount of foam cells accumulation and their apoptosis/necrosis forms necrotic core of unstable plaques. In these plaques, macrophages continue to be the major contributor for lesion advancement and inflammatory response via the secretion of pro-inflammatory mediator [9]. Therefore, intensive research efforts have been focused on targeting macrophages for AS therapy.

Currently, various strategies have been attempted to target pro-atherogenic macrophages with beneficial effects on AS [10, 11], such as altering lipid metabolism, macrophages depletion, macrophages repolarization, promoting macrophage efferocytosis, apoptosis, or emigration. Because of the complexity mechanisms of AS development and progression, the quantitative efficacy of each strategy highly depends on the stage of disease, as well as the preclinical models. For example, macrophages apoptosis is beneficial in early plaque development, while it would give an adverse effect in complex plaques [12]. Because efferocytosis is impaired at advanced stage, apoptosis of macrophages causes the abundant release of lipid content to expand the necrotic core and enhance its thrombogenicity. However, the plaques at different stages of development can be find in an individual AS patient [13]. Therefore, strategies with multiple therapeutic pathways are preferred that can compensate the intrinsic limitations of each other with synergistic effect. In addition, to combat the chronic disease of AS, long-term treatment is usually required for most therapies, and thus especial attention has been paid to plaques targeted treatments with reduced side-effects.

To this end, increasing evidences have demonstrated the potential of nanomedicine as an effective targeting strategy for precise therapy of AS [14–16]. The systemic administrated nanoparticles can be endocytosed by circulating phagocytes for subsequent translocation into atherosclerotic lesions via cellular recruitment and infiltration [17], or passively accumulate into plaques by virtue of the enhanced leakage of injured endothelium and the dysfunctional neovessels in the adventitia [18]. Moreover, nanoparticles can be further equipment with different ligands to directly recognize plaques macrophage through active targeting [19]. With these benefits, various nano-drug delivery systems have been developed for enhanced AS therapy at animal models [15, 20, 21], and notably, those with intrinsic anti-atherogenic activities are particularly attractive as next-generation nanomedicine. For example, synthetic polymers that can competitively block oxidized lipids uptake, attenuate inflammation, and scavenge ROS have been assembled as nanotherapies with definite efficacy [22, 23]. However, most of these polymers require tedious chemical synthesis with uncontrollable reproducibility and large-scale production, and their long-term biosafety in terms of degradation, metabolism, and in vivo clearance still needs to explore for potential clinical translation. Moreover, their capability to load therapeutic drugs with expanded functionalities are also elusive, which however it critical important for the management of AS with complicated syndromes. Therefore, the development of simple, biocompatible, intrinsically active nanoparticles with high drug loading capacity are highly desired in targeted therapy of AS.

**Scheme 1:**
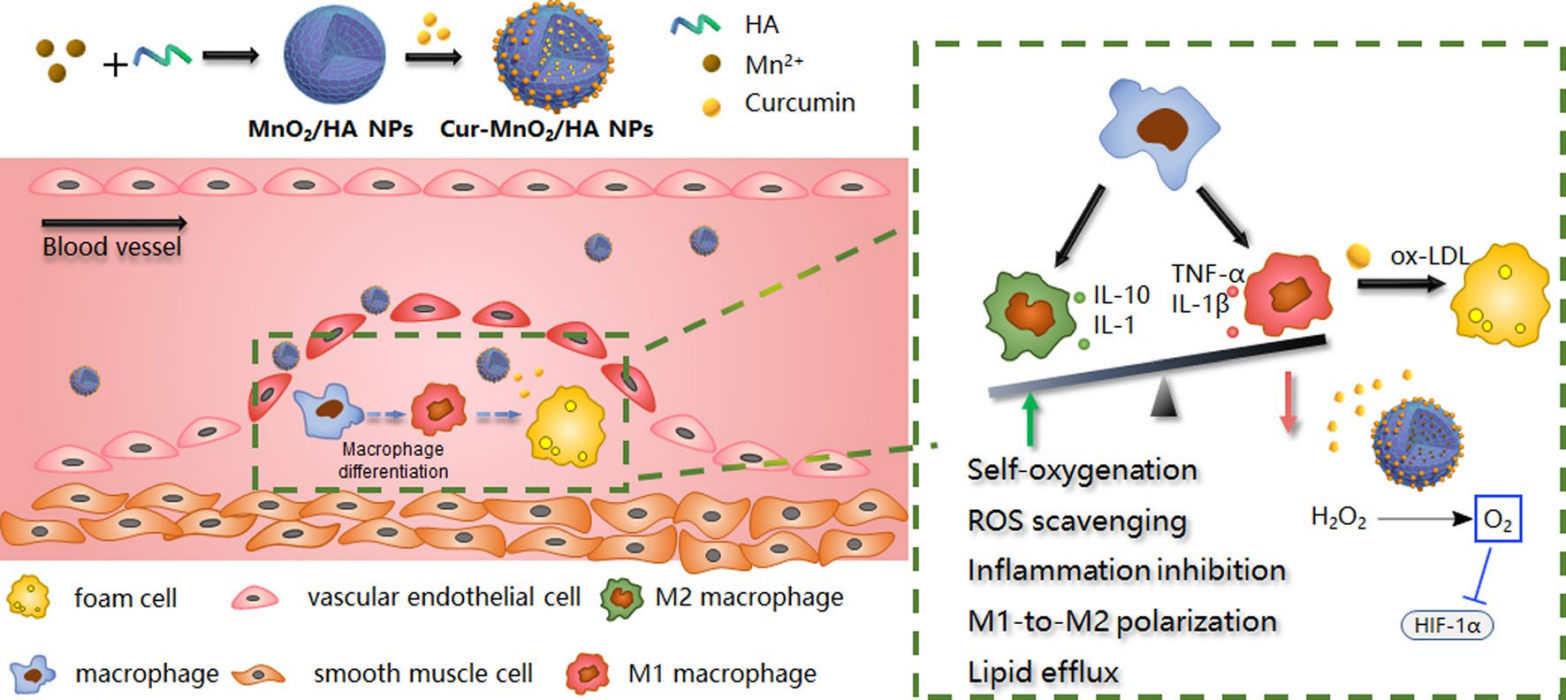
A scheme illustration the preparation of the Cur loaded MnO_2_/HA for targeting delivery in atherosclerotic lesions, and the mechanisms for anti-AS therapy

Herein we reported mesoporous MnO_2_ nanoparticles as a novel nanoplatform for targeted AS therapy (Scheme 1). The MnO_2_ was facilely prepared via a simple mixing/sonication process under mild condition, using hyaluronic acid (HA) as stabilizer. The resulting MnO_2_/HA showed intrinsic catalase mimic activity, which catalyzes the endogenous abundant H_2_O_2_ into O_2_ as self-oxygenation agent to relieve hypoxia in AS site. Moreover, the mesoporous structure of the nanoparticles provided abundant domains for drug loading, which have been demonstrated by using a model drug of curcumin (Cur). Cur, an active polyphenol extracted from the dietary spice turmeric, has been widely used in Asian countries for prevention and treatment of various diseases including AS, owing to its broad anti-inflammatory and anti-oxidant activities [24]. However, its potential clinical application has been restricted by its low solubility in aqueous solution, poor bioavailability, instability and high metabolic rate. Therefore, various nano-drug delivery systems have been reported to address the pharmaceutical limitations of Cur for desirable therapeutic effects [25]. In addition, various co-delivery systems have been developed for synergistic therapy by virtue of multifunctionalities of Cur [26–28]. In our study, Cur was facilely loading into MnO_2_/HA via a simple incubation process, reaching extraordinarily high drug loading capacity up to 54%. The resulting nanomedicine could achieve targeting drug delivery by virtue of its surface HA modification to bind CD44 receptor overexpressed on diseased macrophages surface. Combining the catalytic activity of the nanocarrier and the pharmacological functions of payload Cur, this nanotherapy could effectively attenuate oxidative damage, suppress inflammation, promote M1-to-M2 macrophage repolarization, alleviate hypoxia, as well as inhibit ox-LDL-induced foam cell formation, thus preventing the pathogenesis of AS via multiple mechanisms. After intravenous injection into ApoE^−/−^ mice, the nanomedicine showed significantly prolonged drug circulation, and enhanced accumulation in atherosclerotic lesions, giving rise to robust anti-AS effect with high biosafety. This work provides a functional nanomaterial with ultra-high drug loading capacity to combat pathologically hypoxia diseases, including but not limited to AS.

## Materials and methods

Curcumin (Cur) and rhodamine B were provided by Yuanye Biotechnology Co., Ltd (Shanghai, China), and hyaluronic acid (HA) was purchased from huateng pharmaceutical Co., Ltd. (Jiangsu, China). Manganese dioxide (MnCl_2_) and 2,2-diphenyl-1-picrylhydrazyl (DPPH), human ApoAI and human HDL were obtained from Sigma (Saint Louis, MI, USA). NaOH, ammonium molybdate, Tween 80, H_2_O_2_, 4′, 6-Diamidino-2-phenylindole (DAPI), 2′ 7'-dichlorofluorescin diacetate (DCFDA) were from Sinopharm Chemical Reagent Co., Ltd (Shanghai, China). IFN-γ, IL-4, and 4′, 6-diamidino-2-phenylindole (DAPI) was provided by Solarbio Biotech, Co., Ltd. (Beijing, China). Glutathione (GSH), NaOH, CoCl_2_, dimethyl sulfoxide (DMSO), tween 80 H_2_O_2_ and other common chemical reagents were obtained from Macklin Co., Ltd (Shanghai, China). DCFH-DA was provided by Sigma-Aldrich (St Louis, MO, USA). All mRNA primers were obtained by BioeGene Co., Ltd. (Shanghai, China). Fetal bovine serum (FBS) and dulbecco’s modified Eagle’s medium (DMEM) were from Gibco (Grand Island, NY, USA). Lipopolysaccharide (LPS) was from Biosharp (Hefei, China). Fetal bovine serum (FBS), penicillin, streptomycin, and Dulbecco’s modified Eagle’s medium (DMEM) were from Gibco (Carlsbad, CA, USA). The PCR related agents, including qPCR Detection Kit, TRIzol reagent, and First Strand cDNA Synthesis Kit were from Thermo Fisher Scientific Co., Ltd (MA, USA). The primers of CD206, Arg-1, CD80, iNOS, and housekeeping gene of GAPDH were designed by Qingke Biotech (Changsha, China). NBD-cholesterol was from Ruixi Biotechnology Co., Ltd (Xian, China). Human high-oxidized low density lipoprotein (oxLDL) was purchased from Yiyuan Biotechnologies (China). The assay kits for biochemical indexes in vivo were provided by Jiancheng Bioengineering Institute (Nanjing, China).

### Cells

Murine macrophage line RAW 264.7 was from Xiangya cell center (Changsha, China). The cells were cultured in DMEM media at 37 °C supplemented with 1% penicillin–streptomycin plus 10% FBS in a humidified atmosphere of 5% CO_2_. The macrophages were treated with 100 ng/mL LPS plus 2.5 ng/mL IFN-γ or 10 ng/mL IL-4 for 24 h to allow M1 and M2 polarization, respectively.

### Animals

All animal procedures and protocols were approved by the Experimental Animal Ethics Committee of Central South University and were performed according to the National Regulations on the Use of Experimental Animals. Male C57BL/6 mice (6–8 weeks) as control and the male apolipoprotein E-deficient (ApoE^−/−^) mice (about 6–7 weeks old) were provided by Cavens Laboratory Animal Co., Ltd. (Changzhou, China). The animal experiments were performed after 7 days of acclimatization.

### Preparation of Cur-MnO_2_/HA NPs

NaOH (80 μL, 1 M) was mixed with hyaluronic acid (4 mL, 5 mg/mL) at room temperature, and MnCl_2_ (40 μL, 20 mg/mL) was rapidly added, followed by sonication for 10 min. The MnO_2_/HA NPs was collected via centrifugation at 20,000 rpm for 10 min. To prepare Cur-MnO_2_/HA NPs, curcumin (100 μL, 10 mg/mL, dissolved in DMSO) was added into 1 mL MnO_2_/HA NPs under vigorous stirring. After 10 min, Cur-MnO_2_/HA NPs was obtained by centrifugation. Rho B loaded nanoparticles were similarly prepared by replacing Cur with Rho B.

### Characterizations of Cur-MnO_2_/HA NPs

The UV–Vis absorbance and FT-IR spectra were measured by UV–Vis spectrophotometer (UV2450, Shimadzu, Tokyo, Japan) and Fourier Transform Infrared Spectroscopy (FTIR) (ALPHA, Bruker, Germany), respectively. The hydrodynamic size and ζ potential were measured by Malvern Zeta Sizer Nano series (Nano ZS, Malvern instruments, Malvern, UK). To explore the colloidal stability, Cur-MnO_2_/HA NPs were dispersed in DMEM complete medium (containing 10% FBS), PBS solution (10 mM, pH 7.4) or water at 37 °C, and the samples were taken at pre-determined timepoints for particle size monitoring. The morphology and elements distribution were determined by TEM–EDX (Titan G2 60e300, FEI, Waltham, MA, USA) and X-ray photoelectron spectroscopy (XPS, ThermoFisher-VG Scientific, ESCALAB250Xi, Madison, Waltham, MA, USA). The loading capacity (LC%) and encapsulation efficiency (EE%) of Cur was calculated according to the following formulas.

1 $$LC{\%}=\frac{Weight \,of\, Cur\, in\, nanoparticles}{Weight\, of\, nanoparticles}\times 100{\%}$$

2 $$EE{\%}=\frac{Weight\, of\, Cur\, in\, nanoparticles}{Weight\, of\, feeding\, Cur}\times 100{\%}$$

To study the Cur release profile, Cur-MnO_2_/HA NPs was suspended in different buffers (pH 7.4 phosphate buffer, pH 7.4 phosphate buffer plus 10 mM GSH, and pH 5.5 phosphate buffer) containing 0.2% tween 80 to maintain the sink condition. At predetermined timepoints, the dissolution media were sampled to measure drug release by measuring Cur. To study the interaction between Cur and MnO_2_/HA NPs, 100 mM probing ligand (NaCl, urea, EDTA, or SDS) was added into Cur-MnO_2_/HA NPs for 1 h incubation, and then the nanoparticles were centrifuged and the supernatant was collected to measure the detached Cur by UV spectrophotometer.

### Catalase-mimic and ROS scavenging activities

To measure the catalase mimic activity, 1 mM H_2_O_2_ was added into MnO_2_/HA NPs, followed by measuring the kinetics of H_2_O_2_ consumption and O_2_ generation. Specifically, H_2_O_2_ concentration was quantified by adding 0.5 mL of ammonium molybdate (40 mg/mL), which could react with H_2_O_2_ to form a yellow complex with UV–Vis absorbance at 350 nm. O_2_ concentration can be directly monitored by using a portable dissolved oxygen meter (JPBJ-609L, INESA Scientific Instrument Co., Ltd., Shanghai, China).

To measure the DPPH scavenging activity, 1 mL fresh prepared DPPH^·^ solution (100 μL/mL) was incubated with different concentrations of Cur-MnO_2_/HA NPs at 30 °C in dark. After 30 min, the UV–Vis absorbance at 517 nm was recorded to calculate DPPH elimination.

Excess amount of superoxide anion (O_2_^·−^) was produced by the xanthine/xanthine oxidase system, and then various concentrations of Cur-MnO_2_/HA NPs was added for 40 min incubation at 37 °C. The residual O_2_^·−^ was quantified by a commercially available test kit (Nanjing Jiancheng Bioengineering Institute, China) to calculate O_2_^·−^ scavenging activity.

Hydroxyl radical (·OH) scavenging activity was measured by using a hydroxyl radical antioxidant capacity (HORAC) activity assay kit (Cell Biolabs, Inc., USA), which is based on the oxidation of a fluorescent probe by ^·^OH. By following the manufacturer’s protocol, ^·^OH scavenging activity of different concentrations of Cur-MnO_2_/HA NPs was explored.

### Cellular uptake

The M1/M2 polarized macrophages were seed in 24-well plates (5 × 10^3^ cells/well), and Rho B labeled nanoparticles were added for 4 h incubation. After removing the cell media, the cells were washed three times with PBS (10 mM, pH 7.4), and then fixed by 4% paraformaldehyde for 20 min, followed by staining the cell nuclei using 2 μg/mL DAPI for 10 min. To explore the contribution of HA-mediated intracellular delivery, the cells were pre-treated with 5 mg/mL HA for 4 h before adding the nanoparticles. The uptake of nanoparticles was observed by using fluorescent microscopy. For quantitative analysis, the uptake was examined by using flow cytometry (FACSVerse, BD, USA). The cells were incubated in 6-well plates at density of 4 × 10^4^ cells/well, and all treatments were the same as described above. After incubating with nanoparticles and washing by PBS, the cells were collected and suspended in PBS for flow cytometry analysis.

### Cell cytotoxicity

Cell cytotoxicity of Cur-MnO_2_/HA NPs was evaluated by MTT assay. Briefly, the M1 macrophages were seed in 96-well plates, and Cur-MnO_2_/HA NPs with different Cur concentrations were added for 24 h incubation. The medium was replaced by 100 μL MTT solution (0.5 mg/mL), followed by 4 h reaction. After discarding the liquid in wells completely, DSMO was added to dissolve formazan crystals, and the UV–vis absorbance at 490 nm was determined by microplate reader (Infinite M200 PRO, TECAN, Austria) to assess the cell viability.

### Intracellular ROS scavenging activity

RAW 264.7 macrophages (10^5^ cells/well) were seeded in 24-well plates, and incubated with free Cur or Cur-MnO_2_/HA NPs (at equivalent Cur concentration of 60 μM) for 2 h. For control groups, the cells were cultured in medium without any treatment. Then, the cells were treated with 100 ng/mL LPS plus 2.5 ng/mL IFN-γ for 24 h, while the negative control was treated with fresh medium again. After washing with PBS, the cells were treated with 10 μM DCFH-DA in serum-free medium for 30 min incubation. The fluorescence images were taken by using fluorescence imaging system (NIKON, Ti-S, Japan), and the quantified intensity was measured by flow cytometry (Accuri C6, BD Biosciences).

### In vitro anti-inflammatory effects

The cells received the same treatments as described above. After LPS plus IFN-γ treatments, various inflammatory cytokines in culture supernatants were measured by ELISA (Neobioscience, China), and the total protein levels were determined by BCA kit (Beyotime, China).

### In vitro anti-oxidative protection effects

RAW 264.7 macrophages (10^5^ cells/well) were seeded in 24-well plates, and incubated with free Cur or Cur-MnO_2_/HA NPs (at equivalent Cur concentration of 60 μM) for 2 h. For control groups, the cells were cultured in medium without any treatment. Then, the cells were treated with 300 μM H_2_O_2_ for 24 h. For negative control, the cells were treated with fresh medium for comparison. To measure DNA damage, the total DNA was collected and purified by using a DNA extraction kit (51304, QIAGEN, German) for concentration quantification. Then, DNA was digested by nuclease P1, followed by adjusting the solution pH to ~ 8 using 1 M Tris–HCl. Afterwards, alkaline phosphatase (10 U per mg DNA) was added for 30 min incubation at 37 °C then boiling for 10 min. The 8-OHdG level was measured using an ELISA kit by following the manufacturer’s instruction (SKT-120, Stressmarq, Canada). To determine lipid damage, the level of 8-iso-PGF2α in cell medium was directly measured using an ELISA kit by following the manufacturer’s instruction (ADI-900-010, ENZO, Switzerland).

### M1-to-M2 polarization of macrophages

For RT-PCR, the RAW 264.7 macrophages (10^5^ cells/well) were seed in 6-wells plates and stimulated with LPS plus IFN-γ for M1 polarization. Then, Cur or Cur-MnO_2_/HA NPs (at equivalent Cur concentration of 60 μM) was added for 24 h incubation. Afterwards, the cells were collected and the total RNA was harvested by TRIzol reagent, and the relative level of macrophage-associated mRNAs were measured by RT-PCR (CFX-Connect, BIO-RAD, USA). For immunofluorescent staining, the cells were accepted the same treatments, and then fixed with 4% paraformaldehyde to allow incubation with primary antibodies at 4 °C overnight. Then, the secondary antibodies (with fluorescence-labeling) were added for 1 h incubation. The cell nuclei were co-stained with DAPI, and then observed and imaged by fluorescence microscope.

### HIF-1α and ABCA1 regulation

To induce HIF-1α upregulation, the cells were incubated with 100 μM CoCl_2_ in serum-free medium for 4 h. Then, the cells were treated with different formulations for 24 h, and lysed in RIPA buffer (Servicebio, China). After quantification of the isolated proteins using BCA protein quantification kit (Servicebio, China), equal amounts of proteins were loaded on 5% SDS-PAGE for separation, and then transferred to PVDF membranes for incubation with rabbit HIF-1α monoclonal antibody (1: 1000, ABclonel) for 3 h. Afterwards, secondary antibodies with horseradish peroxidase were incubated with the membrane for 30 min, and the proteins were visualized by a chemical luminescence kit (Servicebio, China). To measure the APBA1 expression, the cells were subjected with different treatments, and the total RNA was harvested by TRIzol reagent, and the relative level of ABCA1 mRNA was measured by RT-PCR (CFX-Connect, BIO-RAD, USA) according to the manufacturer’s instruction.

### Cholesterol efflux assay

The fluorescent NBD-cholesterol was used to study the cholesterol efflux in macrophages as described previously. The cells were treated with different formulations for 12 h, and the cells without any treatment were used as control. Then, 1 μg/mL NBD-cholesterol was added for another 6 h incubation in presence of 10 μg/mL ApoAI or 50 μg/mL HDL. The cells were washed with PBS and cultured in cell medium for 6 h. Afterwards, the cell medium was collected and centrifuged (13,000 rpm, 5 min) to remove debris, while the cells were lysed by NaOH (0.5 mL, 0.1 M). NBD-cholesterol concentration was quantified by fluorescent microplate reader (Infinite M200 PRO, TECAN, Austria) with Ex = 496 nm, Em = 537 nm, and the percentage of cholesterol efflux was calculated.

### Oil Red O staining the formation of foam cell

The cells were added with 30 μg/mL oxLDL in presence or absence of different formulations for 48 h incubation. For the control group, the cells were treated with cell medium. After washing twice by PBS, the cells were fixed by 4% paraformaldehyde for 20 min, and then washed with water and isopropanol. Afterwards, the cells were stained with Oil Red O for 15 min, followed by rinsing with isopropanol (30 s) and washing by water (twice). The cell nuclei were counter-stained with hematoxylin for 1 min. After washing with water, the lipid droplets in cells were observed using light microscope, and the ORO concentration was quantified by extracting intracellular ORO using isopropanol for UV–vis measurement at 518 nm.

### In vivo pharmacokinetics and biodistribution

The mice were intravenously injected with free Cur solution (dissolved in a mixture solution of DMA: PEG400: 5% dextrose with volumetric ratio of 3:9:8) or Cur-MnO_2_/HA with Cur dose of 10 mg kg^−1^ body weight. At pre-determined timepoints, 400 μL blood samples were collected, and centrifuged for 10 min (4 °C, 5000 rpm min^−1^) to obtain the plasma. Then, the plasma (150 μL) was added with citrate buffer solution (50 μL, pH 3), followed by 3 min incubation. After dilution by adding 1.5 mL methanol with gently whirling, the sample was centrifuged (4 °C, 10,000 rpm min^−1^, 10 min), and the supernatant was collected for Cur quantification by using fluorescent meter at excitation/emission of 420/540 nm (the raw data were presented in Additional file 1: Table S1 and Fig. S1, and a calibration for sample quantification was built in Additional file 1: Fig. S2). To measure the biodistribution, the mice were sacrificed at 6 h post-injection, and the major organs as well as aortas were harvested. After washing and weighting, the samples were homogenized, followed by adding citrate buffer solution (500 μL, pH 3) for vortex. Then, the mixture was diluted by 3 mL methanol with whirling for 10 min. After centrifugation to obtain the supernatant, the Cur concentration in each tissue was measured as described above. All raw data for each sample were shown in Additional file 1: Figs. S3, S4.

### Therapeutic efficacy evaluation in vivo

The ApoE^−/−^ mice were fed with a high-fat diet during the whole experiment to allow the development of AS model, while the control group received normal feeding. After the first month, the model mice were randomly divided into four groups with different treatments via tail vein injection (i.e., saline, MnO_2_/HA, Cur, and Cur-MnO_2_/HA; with equivalent Cur dosage of 20 mg/kg) for every three days. Two months later, the mice were sacrificed, and the aorta was collected and washed with 10% neutral buffered formalin for 30 min for subsequent evaluation. The total aorta and aortic root were stained with ORO, and the plaque area was analyzed by Image-Pro Plus 6.0 software. For histology assay, the artic sections were fixed in 10% neutral buffered formalin, and embedded in paraffin with 4 μm thickness, followed by hematoxylin and eosin (H&E) staining.

### Mechanism studies of the anti-AS efficacy and biosafety evaluation

After various treatment, the serum samples were collected, and levels of MDA, H_2_O_2_, ox-LDL, TNF-α and IL-1β measured by their respectively assay kits (Nanjing Jiancheng Bioengineering Institute, Nanjing, China). The aortas were excised to allow immunofluorescent staining of HIF-1α, ABCA1 mRNA level was measured by RT-PCR. To explore the macrophage polarization, the aortas were pretreated with BSA for deparaffinization, antigen retrieval and blockade, and the sections were incubated with the antibodies against Arg-1 and iNOS. Afterwards, the secondary antibodies with fluorescence labeling were added for 1 h incubation. Then the cell nuclei were co-stained by DAPI, and observed by a fluorescent microscope. For biosafety evaluation, major organs were collected to fix in 4% paraformaldehyde, and stained with H&E for analysis.

### Statistical analysis

All data are expressed as the mean ± standard deviation (SD). Statistical analyses were conducted using the one-way ANOVA test for experiments with multiple groups, and a student’s t-test for data with two groups. Statistical significance was considered at p < 0.05.

## Results and discussion

### Preparation and characterizations of Cur-MnO_2_/HA

**Fig. 1:**
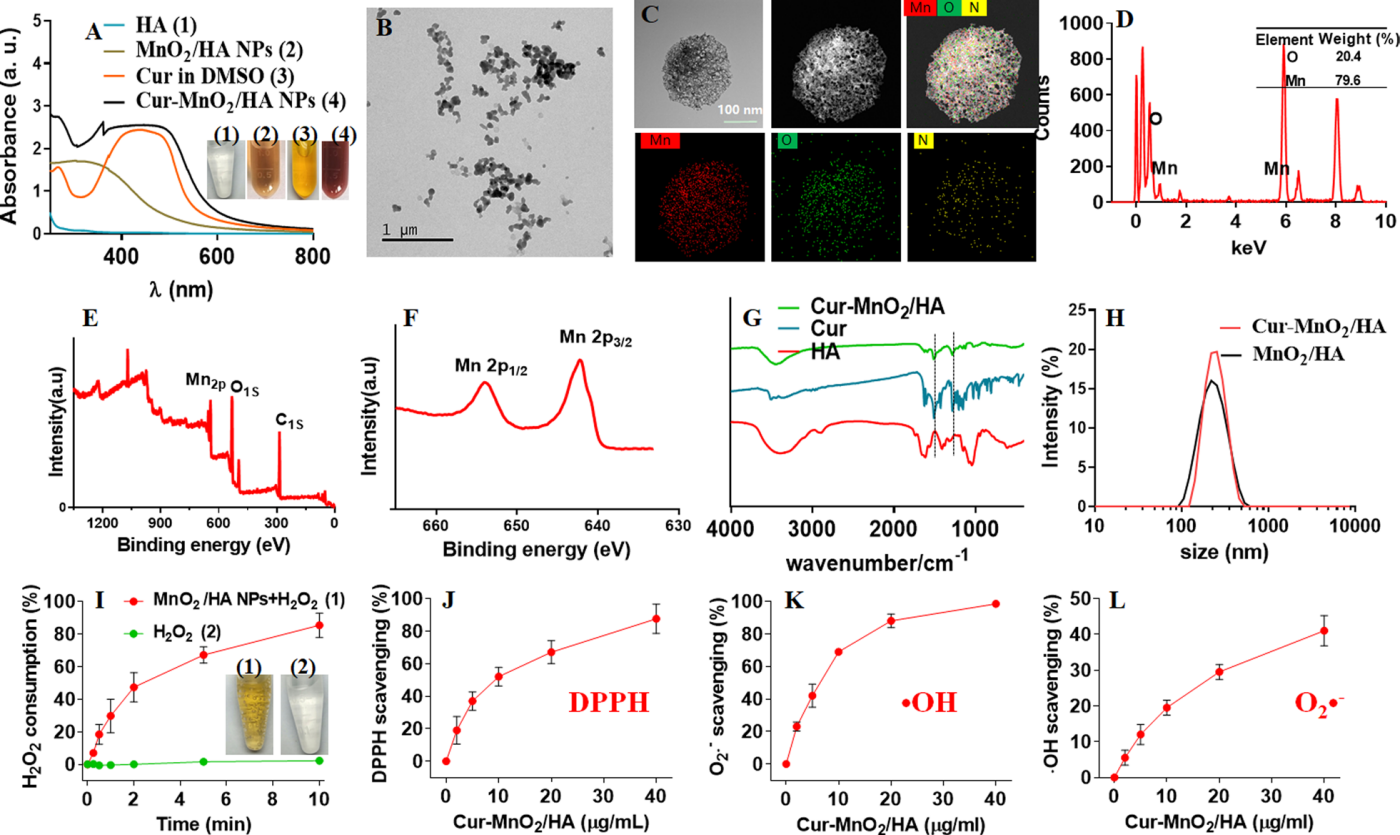
Characterizations of Cur-MnO_2_/HA NPs. **A** UV–vis absorbance spectra of HA, MnO_2_/HA NPs, free Cur, and Cur-MnO_2_/HA NPs. Inset: The appearance of each solution. **B** TEM image, **C** elemental mapping, **D** EDS analysis, **E** XPS spectrum, and **F** Mn 2p XPS spectrum of MnO_2_/HA NPs. **G** FT-IR spectra of HA, free Cur, and Cur-MnO_2_/HA NPs. The characteristic peaks of (1) 1281 cm^−1^ and (2) 1510 cm^−1^ of Cur can be ascribed to δ(CH) of C=CH and ν(C=O), respectively [35]. **H** Dynamic size of MnO_2_/HA NPs, free Cur, and Cur-MnO_2_/HA NPs. **I** Kinetics of H_2_O_2_ consumption and O_2_ generation catalyzed by MnO_2_/HA NPs. Radical scavenging activity of Cur-MnO_2_/HA NPs at different concentrations for **J** DPPH, **K** O_2_^·−^, and **L** ^·^OH

MnO_2_/HA was facilely synthesized via oxidation of Mn^2+^ under basic condition using HA as stabilizer (Scheme 1). Briefly, Mn^2+^ and HA were mixed at pH 14, and the color of the solution rapidly changed to brown within 1 min (Fig. 1A). Such reaction can be easily monitored by UV–Vis, and an absorbance spectrum around 300–400 nm was intensified. This peak can be ascribed to surface plasma band of colloidal manganese dioxide [29], which confirms the formation of MnO_2_ nanoparticles. The nanoparticles showed uniform size distribution in TEM micrograph (Fig. 1B), and from high resolution TEM the nanoparticle exhibited spherical and mesoporous morphology with particle size ~ 200 nm (Fig. 1C). In addition, the SEM characterization was also performed, and consistent result was observed (Additional file 1: Fig. S5). The elemental mapping showed Mn and O signal throughout the nanoparticle, while N signal (originated from HA) was only observed from particle surface, verifying the HA stabilized MnO_2_ nanoparticles. To quantify the surface elemental composition, Energy-dispersive X-ray spectroscopy (EDS) analysis was performed (Fig. 1D), and the surface mainly consisted of Mn and O, accounting for 79.6% and 20.4%, respectively. From X-ray photoelectron spectroscopy (XPS), strong Mn signal was observed (Fig. 1E, F), which showed two characteristic peaks at 642.4 eV and 654.2 eV assigning to Mn (IV) 2P1/2 and Mn (IV) 2p2/3 spin–orbit peaks of MnO_2_ respectively, further confirming the MnO_2_ formation. We further characterized MnO_2_/HA by XRD, while very weak signal was seen with a broad diffraction (Additional file 1: Fig. S6), suggesting the amorphous property of the nanoparticles.

Cur was then loaded into nanoparticles via hydrophobic interaction, and successful Cur loading was evidenced by both UV–Vis and FT-IR spectra (Fig. 1A, G), where the characteristic peaks of Cur were also seen in Cur-MnO_2_/HA. The color of the nanoparticles became much deeper after Cur loading (Fig. 1A, inset), while the dynamic size of particle was almost unchanged to be 230 nm (Fig. 1H). The surface charge, however, became less negative after Cur loading (Additional file 1: Fig. S7). Based on HPLC characterization, the Cur loading capacity was measured to be ~ 54%, which is significantly higher than most other Cur encapsulation nanoparticles [30], due to the mesoporous structure of the nanoparticles. We further explored the binding force by using various probing ligands (including NaCl, urea, EDTA, and SDS), and only SDS could effectively dissociate Cur from nanoparticles (Additional file 1: Fig. S8), demonstrating the critical role of hydrophobic interaction for Cur loading. By virtue of HA stabilization, Cur-MnO_2_/HA achieved excellent colloidal stability under various media with almost unchanged particle size over 24 h incubation, which is highly important for biological applications (Additional file 1: Figs. S9, S10, Table S2). Note that the nanoparticles showed sustained release profile at physiological mimic condition (i.e., PBS buffer, pH 7.4), while a triggered drug release was observed in response to intracellular stimuli (such as endosomal acid pH and GSH) (Additional file 1: Fig. S11). Therefore, Cur can be stably adsorbed on Cur-MnO_2_/HA during in vivo circulation, while rapidly liberate from nanoparticles after being delivered into target cells to exert biological functions, thus decreasing the administration frequency and minimizing potential side-effects.

Besides barely acting as a drug loading carrier, another purpose of using MnO_2_/HA is its potential as catalase-mimic nanozyme for self-oxygenation via decomposition of H_2_O_2_ [31], which is rather beneficial for AS therapy. To substantiate this, MnO_2_/HA was mixed with H_2_O_2_, and H_2_O_2_ concentration was dynamically monitored by using Góth method as described in our previous work [32]. H_2_O_2_ was rapidly consumed in 10 min, accompanied by the appearance of large number of bubbles in test-tube (Fig. 1I). To confirm the oxygenation, we also measured the O_2_ production by using portable dissolved oxygen meter. In line with H_2_O_2_ consumption, a fast O_2_ generation kinetics was observed, demonstrating high catalytic activity of MnO_2_/HA (Additional file 1: Fig. S12). After Cur loading, the nanoparticles were further equipped with ROS scavenging activity endowed by Cur [33]. We first evaluated the ROS scavenging capability by using DPPH, a commercial available free radical compound, and such radical was eliminated in a concentration-dependent manner (Fig. 1J). At 40 μg/mL Cur, DPPH was completely cleared, suggesting high activity of Cur-MnO_2_/HA. Inspired by this, we further explored the broad spectrum of ROS scavenging activity by testing other physiologically relevant radicals, including superoxide anion (O_2_^·−^) and hydroxyl radical (^·^OH) (Fig. 1K, L). Similarly, we observed a general trend of ROS scavenging at higher Cur concentrations. Collectively, these results demonstrated multi-antioxidative activities of Cur-MnO_2_/HA, and this function can be originated from the phenolic hydroxyl groups in Cur structure [34].

### Targeting delivery towards M1 macrophages

**Fig. 2:**
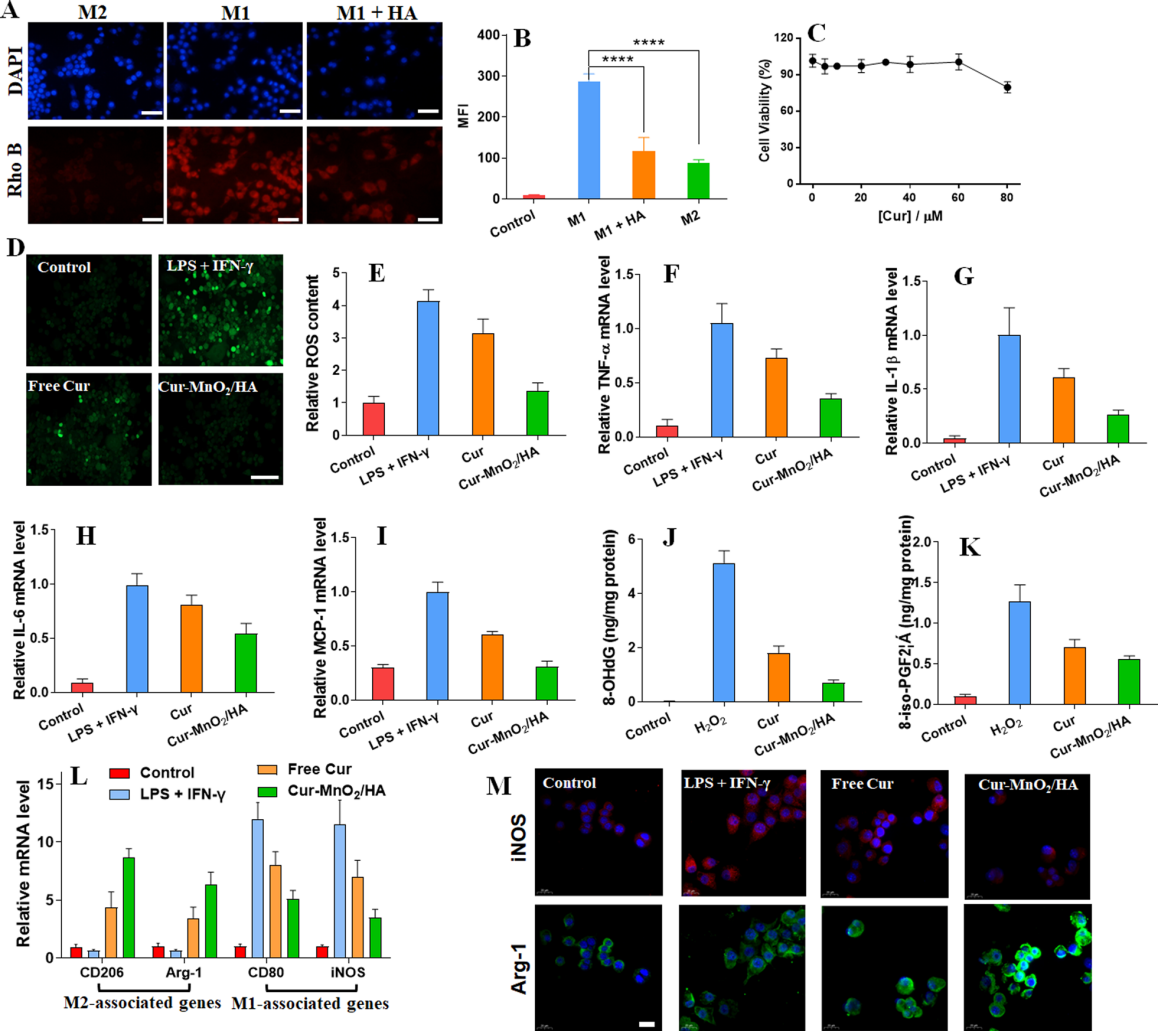
Cellular uptake and biological effects of Cur-MnO_2_/HA NPs in macrophages. **A** Fluorescent images and **B** flow cytometry analysis of Cur-MnO_2_/HA NPs uptake by M1/M2 macrophages for 4 h incubation. To compete CD44 binding, the cells were pretreated with 5 mg/mL free HA for 4 h. Scale bar = 50 μm. **C** Cell viability of the macrophages after incubating with different concentrations of Cur-MnO_2_/HA NPs for 24 h. **D** Fluorescent images and **E** fluorescent intensity quantified by flow cytometry to probe the ROS level (DCFH-DA probe) in macrophages stimulated by LPS + IFN-γ for 4 h, and further treatment with different formulations. Scale bar = 50 μm. The inflammatory cytokines of **F** TNF-α, **G** IL-1β, **H** IL-6, **I** MCP-1 secreted by RAW 264.7. The cells were pre-treated with medium or different formulations, followed by stimulation with IFN-γ + LPS for 24 h. Expression levels of **J** 8-OHdG and **K** 8-iso-PGF2α measured by ELISA. The cells were pre-treated with different formulations for 2 h, and then incubated with H_2_O_2_. **L** The relative expression of M1 and M2 associated genes in RAW 264.7 after different treatments. **M** Immunofluorescence staining of iNOS and Arg-1 in RAW 264.7 after different treatments. Scale bar = 20 μm

M1 macrophages plays critical roles in the development of AS, which has been recognized as important target for site-specific delivery of nanomedicines. We then examined cellular uptake of Cur-MnO_2_/HA NPs in M1 macrophages. For convenient observation, the nanoparticles were labeled with a red fluorescent rhodamine B (Rho B), and the cell nuclei were stained blue with DAPI. The macrophages were pretreated with lipopolysaccharide (LPS) plus interferon-γ (INF-γ) to allow M1 polarization, while the cells with interleukin-4 (IL-4) treatment for M2 polarization were used as control. After 4 h incubation, the red fluorescence was much brighter in M1 macrophages than M2 macrophages (Fig. 2A), indicating a cell selective internalization of the nanoparticles. This can be attributable to the surface HA modification of the nanoparticles, which endows the specific recognition towards CD44 over-expressed M1 macrophages [36]. To validate this, we further pre-treated the M1 macrophages with free HA to compete CD44 binding, and in this case the fluorescence inside cells weakened obviously, demonstrating the crucial contribution of CD44-mediated intracellular delivery. Further, the flow cytometry was performed for fluorescence quantification, in which M1 macrophages displayed ~ threefold higher intensity than M2 macrophages (Fig. 2B), confirming the targetability of the nanoparticles. However, the pre-treatment of HA largely abolished this targeting effect, consistent with the above observation. Therefore, Cur-MnO_2_/HA NPs could be selectively endocytosed by M1 macrophages for targeted therapy.

### Anti-oxidative, anti-inflammatory and M1-to-M2 re-polarizing activity of Cur-MnO_2_/HA NPs in macrophages

Having confirming the targeting intracellular delivery, we next explored the functions of nanosystem inside cells. The payload Cur have been reported to have a broad anti-oxidant and anti-inflammatory activities for various diseases treatment [37], and here we aimed to employ these properties to combat AS. AS is featured with dysregulated generation of ROS with oxidative stress, which damages the cells and tissue and exacerbate the disease via triggering inflammation. To combat this, we loaded Cur into nanoparticles as ROS scavenger, and the ROS scavenging activity were tested by using macrophage with LPS plus IFN-γ pre-treatment. Note that both MnO_2_/HA and Cur-MnO_2_/HA displayed low cytotoxicity towards macrophages and mouse vascular smooth muscle cells (MOVAS), with > 90% cells viable at equivalent Cur concentration of 60 μM after 24 h incubation (Fig. 2C, Additional file 1: Fig. S13), and such concentration was employed for subsequent studies. We employed DCFH-DA probe to visualize the intracellular ROS level, which can be converted into green fluorescent DCF under oxidative condition for ROS monitoring by fluorescent signal. The macrophages with LPS + IFN-γ stimulation exhibited high ROS level inside cells, while the intensity was obviously attenuated with free Cur treatment, and the signal became even weaker after Cur-MnO_2_/HA NPs treating (Fig. 2D). Quantitative analysis by flow cytometry further confirmed that ROS in stimulated macrophages can be effectively scavenged by Cur-containing formulations, especially for Cur-MnO_2_/HA NPs (Fig. 2E), likely due to NPs-mediated intracellular delivery.

It is known that the intracellular accumulated ROS could activate a series of signal transduction cascades, which stimulates inflammation to promote the development of AS [38]. Cur-MnO_2_/HA displayed excellent ROS scavenging activity, and we next explored whether such capability could benefit for inflammation inhibition. The macrophages with LPS plus IFN-γ treatment showed significantly up-expression of various inflammatory factors, including TNF-α, IL-1β, IL-6, and MCP-1, indicating M1 polarization with pro-inflammatory properties (Fig. 2F–I). Preincubation of free Cur could suppress the expression of these cytokines to some extent, and notably, strongly inhibition of these cytokines was observed upon treatment of Cur-MnO_2_/HA, suggesting the robust anti-inflammatory activity of the nanoparticles. Therefore, Cur-MnO_2_/HA could effectively decrease intracellular ROS level, and subsequently attenuate inflammation in macrophages for AS therapy. In addition to the direct ROS scavenging activity, the intrinsic anti-inflammatory activity of Cur would also contribute to such cytokines inhibition effect.

Another adverse effect of excessive ROS production is its direct cell damage via oxidation of intracellular proteins, lipids, and DNA [39]. With ROS scavenging activity, Cur-MnO_2_/HA was expected to protect biomolecules from ROS-induced oxidative injury. To explore this, we measured 8-hydroxy-2′-deoxyguanosine (8-OHdG) and 8-isoprostaglandin F2α (8-iso-PGF2α) in macrophages, which can be used as the markers of DNA and lipid oxidative damage, respectively [40, 41]. To induce oxidative damage, the cells were pretreated with H_2_O_2_, and a remarkable increase of both 8-OHdG and 8-iso-PGF2α was observed (Fig. 2J, K). Consistent with the above ROS scavenging activity, both free Cur and Cur-MnO_2_/HA could dramatically decrease the levels of these markers, and specifically, Cur-MnO_2_/HA achieved much better efficacy. Therefore, Cur-MnO_2_/HA is promising nanoplatform to protect macrophages from oxidative damages.

Macrophages are mainly differentiated into two phenotypes of M1 (pro-inflammatory) and M2 (anti-inflammatory) macrophages. In AS site, macrophages are predominately polarized into M1 macrophages and participate in the occurrence and development of the lesions [42]. Therefore, reprograming M1 towards M2 macrophages has become an attractive therapeutic strategy to manage RA [43]. Various anti-oxidant and anti-inflammatory nanomedicines have been developed to switch M1 macrophages into M2 phenotypes by virtue of the critical roles of ROS and cytokines in macrophages polarization [3]. With the ROS scavenging and anti-inflammatory activity, we next tested the capability of Cur-MnO_2_/HA for macrophages regulation. To do this, phenotype-related mRNA level was analyzed (Fig. 2L). After LPS + IFN-γ pretreatment, the expression of M1 markers (i.e., CD80 and iNOS) significantly up-regulated, accompanied by the decrease of M2 markers (i.e., CD206 and Arg-1), indicating successful stimulation for M1 polarization. Upon incubation with free Cur, the M1 markers were alleviated to some extent, as well as the increase of M2 markers. Specifically, Cur-MnO_2_/HA showed much better M1-to-M2 phenotypic transition efficacy, attributable to the intracellular Cur delivery by the nanoparticles. In addition, the catalase-mimic activity of Cur-MnO_2_/HA was able to inhibit HIF-1α via self-supplied oxygenation (vide infra), which also contributes to the regulation of macrophage re-polarization [44]. For direct observation, we visualized the cell phenotype by immunofluorescence staining of iNOS and Arg-1 with red and green, respectively. Consistent with the above results, the fluorescence of iNOS strongly inhibited while the Arg-1 fluorescence became intensified after Cur and Cur-MnO_2_/HA treatment (Fig. 2M). Therefore, Cur-MnO_2_/HA treatment could effectively induce M1-to-M2 re-polarization of macrophages for AS therapy.

## Promoting cholesterol efflux to inhibit foam cell formation

**Fig. 3:**
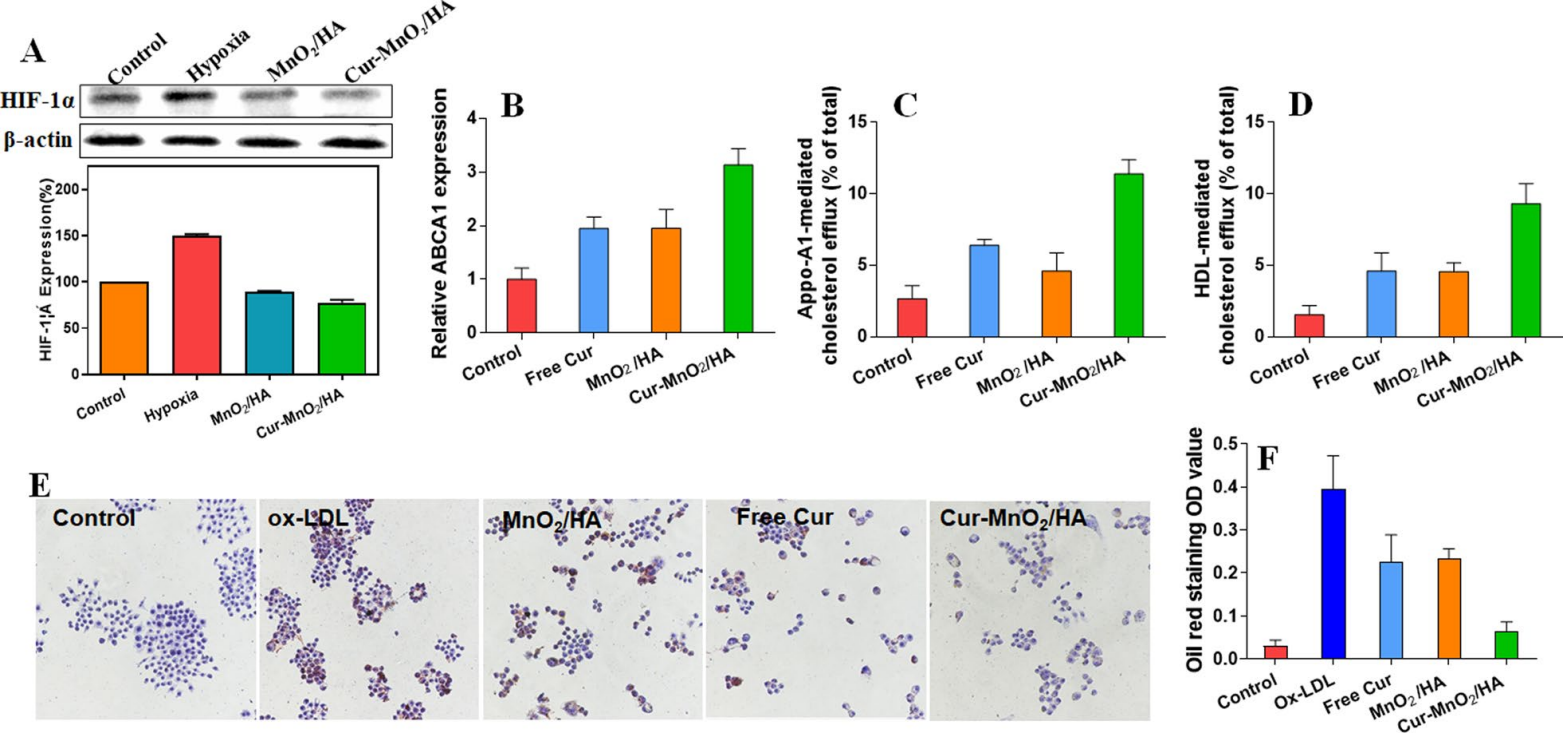
**A** Western blot analysis the expression of HIF-1α protein in macrophages with different treatments, and the quantified results. **B** Relative expression of ABCA1 mRNA after different treatments. NBD-cholesterol-loaded macrophages treated with different formulations to evaluate their effects on **C** Apo-A1-mediated and **D** HDL-mediated cholesterol efflux. **E** Microscopic imaging showing the activity of different formation to inhibit ox-LDL-mediated foam cell formation in macrophages, and **F** the quantification of ORO staining in foam cells derived from macrophage after different treatments

After demonstrating the biofunctions of Cur, we then studied the potential role of MnO_2_/HA as catalase mimic nanozyme for AS therapy. Foam cell formation in intima site is the hallmark of the early development of atherosclerotic lesion, in which lipid metabolism disorder plays critical role during this process [45]. Normally, the lipid (mainly the LDL) is swallowed by macrophage and hydrolyzed into free cholesterol (FC) in lysosomes. Part of the FC then converts into cholesteryl ester (CE), while rest is removed from cell via efflux pathways, such as transporter of ABCA1, thus resulting in lipid metabolism hemostasis. However, under hypoxic condition of AS site, the accumulated lipid level was significantly higher in macrophages, which is partially attributed to the up-regulation of HIF-1α. The overexpression of HIF-1α could inhibit FC efflux in macrophages via suppressing ABCA1, thus promoting the formation of foam cells [8]. Fortunately, the MnO_2_/HA carrier was capable to catalyze the decomposition of H_2_O_2_ into O_2_ for hypoxia alleviation. To validate this function inside cells, we first treated the cells with CoCl_2_ (100 μM) to upregulate intracellular HIF-1α level [46]. Under hypoxia mimic condition, the HIF-1α expression significantly increased, while both Cur-MnO_2_/HA NPs and MnO_2_/HA NPs (without Cur loading) could effectively down-regulate HIF-1α via self-oxygenation to reverse hypoxia (Fig. 3A), which is beneficial for rescuing the lipid metabolism disorder in foam cells.

After confirming the HIF-1α suppression effect, we further explored the subsequent effect of ABCA1 regulation. Without Cur loading, MnO_2_/HA NPs achieved a notable increase of ABCA1 expression, which can be ascribed to its HIF-1α inhibition activity (Fig. 3B). Interestingly, free Cur could also raise the ABCA1 level with ~ twofold enhancement, which is in a good agreement with previous work that Cur prevents AS through regulation lipoprotein cholesterol metabolism [24]. After treating with Cur-MnO_2_/HA NPs, a further increase of ABCA1 (~ threefold) was observed, indicating a synergistic effect between Cur and HIF-1α suppression effect of MnO_2_/HA NPs. ABCA1 is an important cholesterol efflux pump that relieves the lipid burden inside cells. With significant ABCA1 upregulation, we next investigated the cholesterol transport in macrophages by examining both Apo-A1 and HDL mediated cholesterol efflux (Fig. 3C, D). In line with ABCA1 expression, both free Cur and MnO_2_/HA NPs boosted the cholesterol transportation, and Cur-MnO_2_/HA NPs showed the best efficacy. Therefore, Cur-MnO_2_/HA NPs could diminish lipid accumulation in macrophages for AS therapy via regulating the ABCA1.

Having demonstrated the cholesterol efflux effect, we finally examined the activity of the nanoparticles to inhibit foam cell formation, the hallmark of AS. Excess uptake of ox-LDL and over-accumulation of lipid is the key step for the formation of foam cells, and based on Oil Red O (ORO) staining, substantial lipid droplets were observed in macrophages after incubation with 30 μg/mL ox-LDL, indicating the formation of foam cells. In presence of free Cur or nanoparticles, by contrast, the amount of lipid droplets obviously decreased (Fig. 3E). We quantified the intracellularly deposited ORO (Fig. 3F), in which the efficacy to suppress foam cell formation was in order of Cur-MnO_2_/HA NPs > free Cur ≈ MnO_2_/HA NPs. This result demonstrated activity of the nanoparticles to attenuate the formation of foam cells from macrophages, as well as the synergistic effect of free Cur and its cargo MnO_2_/HA.

## Targeted AS therapy in ApoE^−/−^ mice

**Fig. 4:**
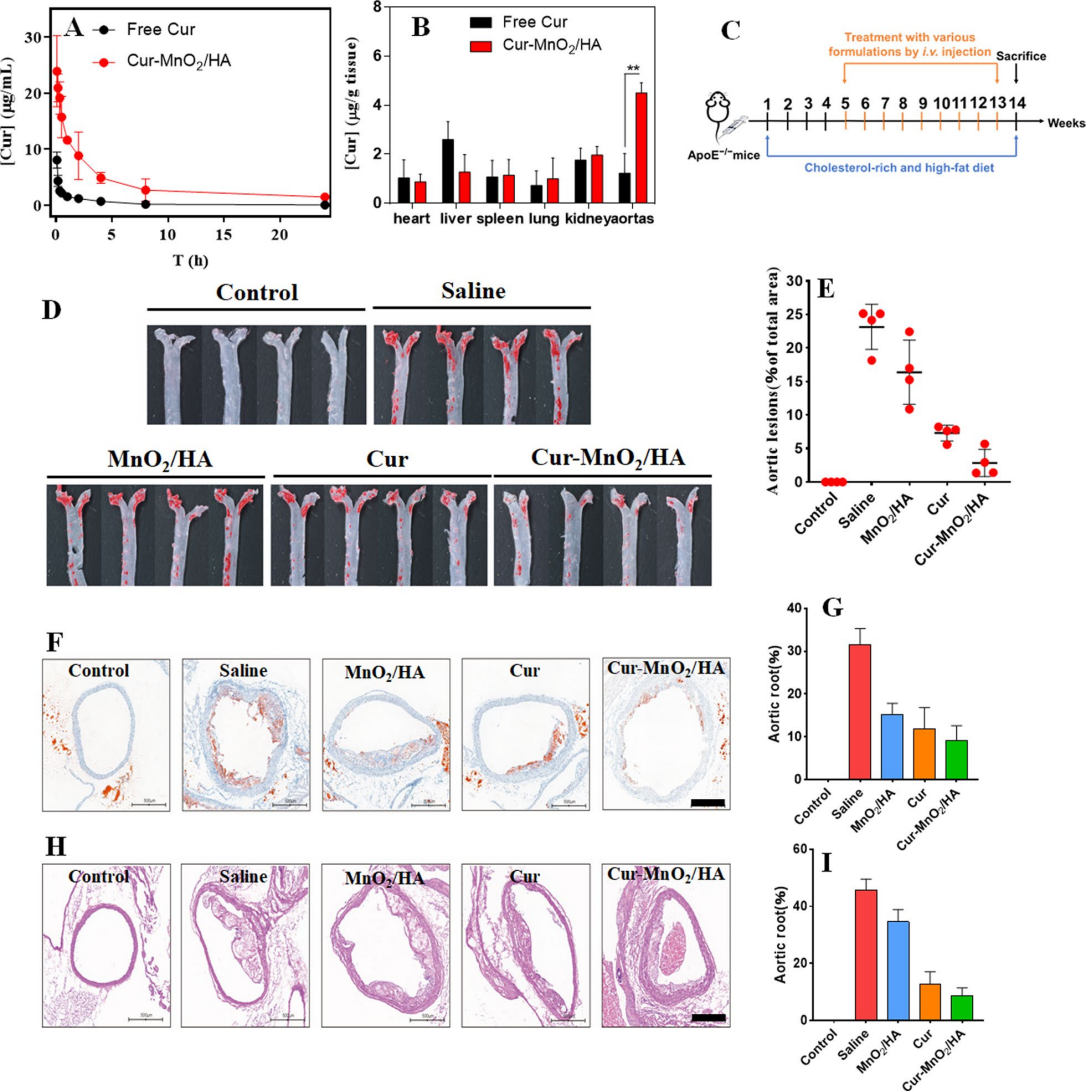
**A** Plasma Cur concentration versus time after intravenous injection of free Cur and Cur-MnO_2_/HA. **B** Biodistribution of Cur at 6 h post-injection of free Cur and Cur-MnO_2_/HA. **C** Schematic showing the treatment process of ApoE^−/−^ mice with various formulations. **D** Representative photographs of ORO-stained aortas from mice after different treatments. **E** Quantification of the lesion area in aortas. **F** ORO-stained cryosections of the aortic root, and **G** the quantitative analysis. **H** Representative H&E images of aortic root sections, and **I** quantification of necrotic core area to plaque area. Scale bar, 500 μm

Having confirmed the functionalities of the NPs at cellular level, we next explored the in vivo performances. First, the pharmacokinetics profile was studied by directly measuring blood Cur concentrations using apolipoprotein E-deficient (ApoE^−/−^). Upon intravenous administration of free Cur, the drug was rapidly cleared with almost undetectable drug content at 8 h post-injection (Fig. 4A). Such rapid elimination presents one critical limitation for clinical applications, which requires frequent administration. Cur-MnO_2_/HA, by contrast, showed much higher serum availability at each timepoint, thus achieving significantly pro-longed circulation half-life (by sixfold). Therefore, the Cur-MnO_2_/HA could effectively improve the bioavailability of Cur by minimizing rapid clearance. We then measured the biodistribution of the drug, and specifical attention has been paid to the aortas from atherosclerotic mice. Different organs were extracted after 6 h injection, and Cur concentration was measured. In general, free Cur and Cur-MnO_2_/HA displayed quite similar distribution in normal organs except liver (Fig. 4B), which is reasonable since liver is the major organ to sequester the administered nanomaterials for subsequent in vivo clearance [47]. Specifically, the Cur-MnO_2_/HA achieved significantly higher drug concentration than free Cur at aortas with atherosclerotic lesions (~ 3.5-fold higher), suggesting the targeting efficiency of the nanoparticles towards AS plaques. This accumulation effect can be ascribed to the leaky endothelial junctions within atherosclerotic vessel and transcellular migration [48], as well as the active recognition of HA modified nanoparticles towards plaque macrophages [49].

After demonstration the targetability in vivo, we then explore the therapeutic efficacy of the nanoparticles. The AS model was developed by feeding the ApoE^−/−^ mice with high-cholesterol-diet (HCD) for 3 months, and the drug intervention started after 1-month HCD treatment for prophylactic therapy, since it is known that ApoE^−/−^ mice begin to develop atherosclerotic plaques at artery bifurcations after 1 month feeding with HCD (please see the detailed treatment scheme in Fig. 4C). Each formulation was administrated via intravenous injection every three days for 2-month treatment, and then the entire aortas was harvested for ORO-staining. Compared with normal-chow-diet (NCD) mice control, the HCD mice with saline injection showed large ORO-positive areas (Fig. 4D), confirming the AS development. By contrast, considerable decrease of atherosclerotic lesion area was observed for all drug/nanoparticles-treating groups. Among then, the best outcome was achieved for Cur-MnO_2_/HA. We also quantified this result by measuring the percentage of average plaque area, in which the efficacy was in order of Cur-MnO_2_/HA > free Cur > MnO_2_/HA.

The aortic root is considered as the initial site of atherosclerotic lesions, where the plaque formation is homogenous and consistent. We therefore collected the samples from this position for histology analysis. Consistent with the above observation, lipid content was obviously reduced upon treatment with different formulations, in which the most significantly result was found in Cur-MnO_2_/HA, displaying 28% decrease in lipid accumulation. The samples were further analyzed by hematoxylin/eosin (H&E) staining to examine the atherosclerotic plaques and the components, in which the saline group showed a large lesion area largely consisting of acellular and lipid-rich necrotic cores. With different formulations treatment, in contrast, the lesion area considerably reduced with substantial regression, especially for Cur-MnO_2_/HA group. Collectively, these results substantiated that Cur-MnO_2_/HA is a highly efficacious nano-platform to ameliorate AS.

### Mechanisms for AS therapy in ApoE^−/−^ mice

**Fig. 5:**
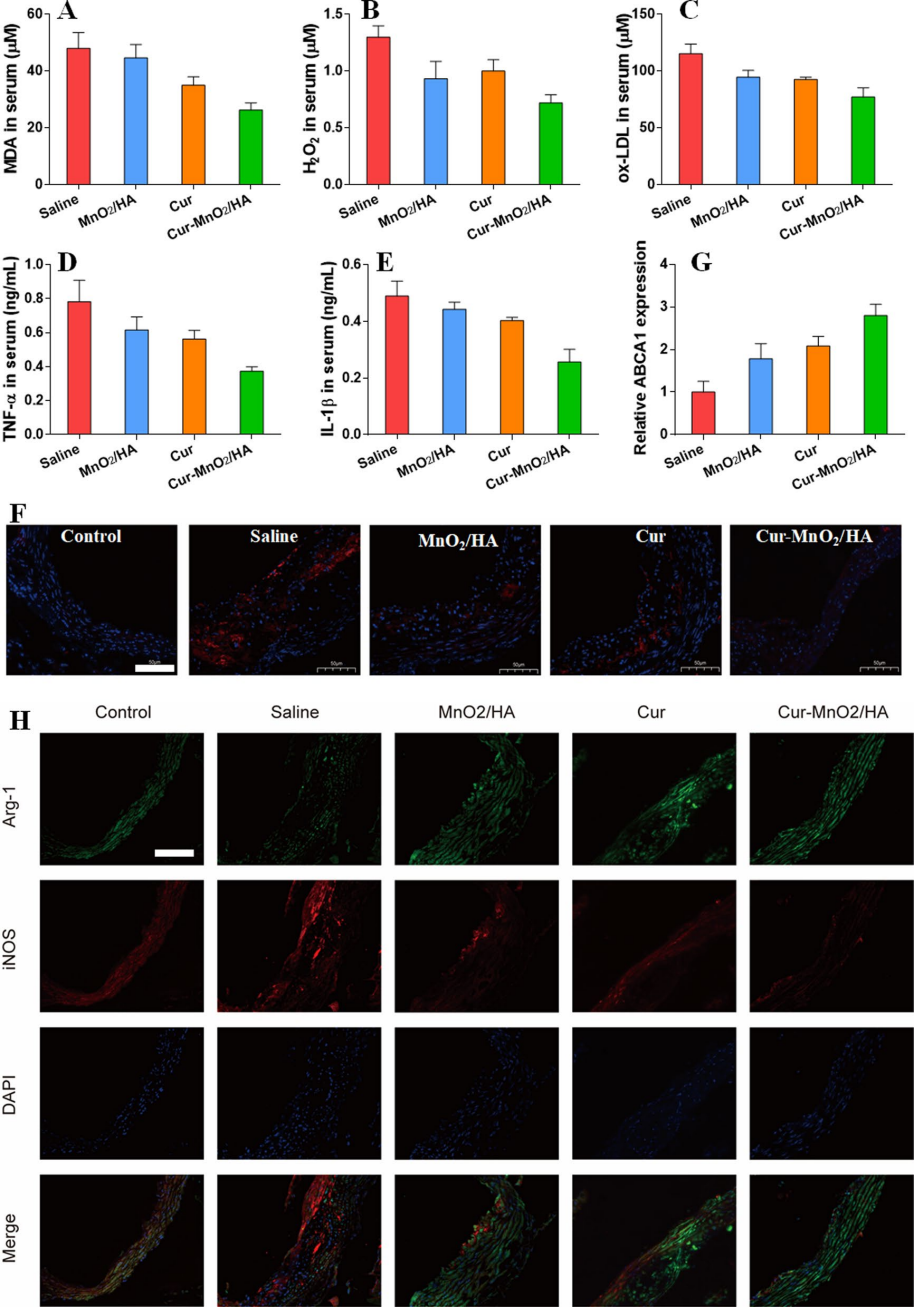
The serum level of **A** MDA, **B** H_2_O_2_, **C** ox-LDL, **D** TNF-α, **E** IL-1β. **F** The immunofluorescence staining of HIF-1α in aortic root of mice after various treatments. Scale bar, 50 μm. **G** Quantification of ABCA1 expression in aortic root by RT-PCR. **H** The immunofluorescence staining of Arg-1 and iNOS in aortic root of mice after various treatments. Scale bar, 50 μm

With satisfactory efficacy in vivo, the nanoparticles were then explored for therapeutic mechanisms. At solution/cell level, the nanoparticles have been demonstrated with multi-functionalities for AS therapy, and these functions were tested side-by-side in vivo. First, the oxidative stress was evaluated by measuring malondialdehyde (MDA), the biomarker of oxidative damage, and H_2_O_2_. After injection with different formulations, the MDA level and H_2_O_2_ concentration were significantly reduced (Fig. 5A, B), suggesting the attenuation of the oxidative stress by virtue of the ROS scavenging activity of free Cur and the nanoparticles. In line with this result, the serum ox-LDL was also inhibited (Fig. 5C). Moreover, the representative pro-inflammatory cytokines were effectively suppressed, including TNF-α and IL-1β, confirming the anti-inflammatory activity of the nanoparticles (Fig. 5D, E).

The atherosclerotic lesions were also collected for pathological analysis. Owing to the self-oxygenation activity of the nanoparticles, HIF-1α level can be effectively controlled by the nanoparticles, as evidenced by the immunofluorescence assay (Fig. 5F). HIF-1α inhibition is advantageous for regulation the lipid metabolism, and we next measured the expression of ABCA1, the key effector for lipid transportation. ABCA1 expression elevated after treatment with different formulations (Fig. 5G), and the highest ABCA1 level was seen in Cur-MnO_2_/HA, due to the co-contribution of Cur and HIF-1α inhibition effect of MnO_2_/HA. Given the ROS scavenging and inflammation inhibiting activities of the nanoparticles, we further explored the phenotypic alteration of lesion macrophages by immunofluorescent staining of Arg-1 (M1 marker) and iNOS (M2). Compared with normal mice, the model mice showed strong fluorescence of M1 marker in AS site, attributable to the pathological polarization of the macrophages. With different treatments, however, the Arg-1 fluorescence weakened, accompanied by the intensified of iNOS fluorescence. As expected, Cur-MnO_2_/HA achieved the most obvious M1-to-M2 phenotypic transition, which could explain the best efficacy for AS therapy. Collectively, these results demonstrated the anti-oxidant and anti-inflammatory activities of Cur-MnO_2_/HA to switch M1-to-M2 repolarization, as well as hypoxia alleviation via self-oxygenation to recover ABCA1 expression, all of which contribute to the AS therapy. Finally, the biocompatibility of the nanosystem was evaluated. Based on the H&E staining, none of the major organs showed any pathological change after a period of 2 months treatment (Additional file 1: Fig. S14), indicating the high biosafety of Cur-MnO_2_/HA for long-term administration.

## Conclusion

In summary, we designed and fabricated a multi-functional nanomedicine to realize macrophage-targeting AS therapy. The MnO_2_/HA was simply prepared and systematically characterized, which possessed intrinsic catalase-mimic activity for self-oxygenation. Importantly, its mesoporous structure is rather advantageous for drug encapsulation, giving an opportunity to load large amount of Cur. Because of surface HA modification, Cur-MnO_2_/HA was able to selectively internalize into M1 polarized macrophages, and exerts a series of therapeutic functions. Specifically, Cur could scavenge a broad spectrum of ROS and suppress the excretion of cytokines, which in turn switch macrophages into M2 phenotype. The catalytic carrier, on the other hand, increased the ABCA1 expression by suppressing HIF-1α, and thus promoted the cholesterol transport to inhibit foam cell formation. Finally, the nanosystem has been applied to ApoE−/− mice, demonstrating its improved pharmacokinetic performance, active targetability, as well as excellent anti-AS efficacy. Overall, this work provides a biocompatible nanomedicine as promising candidate for AS therapy. Given the broad theranostic applications of manganese-based nanomaterials, our MnO_2_/HA with intrinsic catalytic activity and excellent drug loading capacity may pave the way to design smart nano-systems for diagnosis and treatment of various diseases with great clinical translation potential.

## Supplementary Information

**Additional file 1.** Additional information includes raw data for Cur quantification in vivo, SEM image and XRD spectra of MnO2/HA, ζ potential of nanoparticles, Cur adsorption mechanism, colloidal stability of nanoparticles, drug release profiles, catalase-mimic activity of the nanoparticles, cytotoxicity assay, and H&E staining of the major organs after treatments.

## Data Availability

The data used to support the findings of this study are available from the corresponding author upon reasonable request.
