# Supplementary material for: Self-oxygenation mesoporous MnO2 nanoparticles with ultra-high drug loading capacity for targeted arteriosclerosis therapy
Source: J Nanobiotechnology. 2022 Feb 19;20:88. doi: 10.1186/s12951-022-01296-x (PMC8858544; doi:10.1186/s12951-022-01296-x)
Supplement: Supplementary file 1 — Additional file 1. Additional information includes raw data for Cur quantification in vivo, SEM image and XRD spectra of MnO2/HA, ζ potential of nanoparticles, Cur adsorption mechanism, colloidal stability of nanoparticles, drug release profiles, catalase-mimic activity of the nanoparticles, cytotoxicity assay, and H&E staining of the major organs after treatments. [file 12951_2022_1296_MOESM1_ESM.docx]

**Self-oxygenation** **Mesoporous** **MnO_2_ Nanoparticles with Ultra-High Drug Loading Capacity for targeted Arteriosclerosis Therapy**

Weidong Sun^1#^, Yiyan Xu^1#^, Ye Yao^2^, Jie Yue^2^, Zhen Wu^1^, Haocheng Li^1^, Guanghui Shen^1^, Yan Liao^3^, Haiyang Wang*^1^, and Wenhu Zhou*^3^

^1^. Department of Vascular and Interventional Surgery, The First Affiliated Hospital of Harbin Medical University，Harbin，Heilongjiang，150001，China

^2.^ Department of Vascular Surgery，The First Affiliated Hospital of Guangzhou Medical University，Guangzhou，Guangdong，510120，China

^3.^ Xiangya School of Pharmaceutical Sciences, Central South University, Changsha, Hunan, 410013, China

[^#^] These authors contributed equally to this work.

*Email: [haiyanghmu@163.com](mailto:haiyanghmu@163.com); [zhouwenhuyaoji@163.com](mailto:zhouwenhuyaoji@163.com)

**Table S1.** The raw data of calibration curve for Cur quantification.

| Cur (μg/mL) | Fluorescence intensity at 540 nm | | | |
| --- | --- | --- | --- | --- |
|  | Sample 1 | Sample 2 | Sample 3 | Mean |
| 0 | 0 | 0 | 0 | 0 |
| 0.25 | 0.645 | 0.651 | 0.679 | 0.645 |
| 0.5 | 1.171 | 1.1 | 1.101 | 1.124 |
| 1 | 1.843 | 1.918 | 1.851 | 1.871 |
| 2.5 | 4.738 | 4.772 | 4.671 | 4.727 |
| 5 | 9.253 | 9.745 | 10.2 | 9.733 |
| 10 | 16.7 | 15.87 | 16.26 | 16.277 |

**Table S2.** Representative raw data of dynamic zeta potential for Cur-MnO_2_/HA NPs in various media.

| Time (h) | Zeta potential (mV) | | |
| --- | --- | --- | --- |
|  | H_2_O | PBS | DMEM |
| 0 | -24.33±2.12 | -26.7±0.37 | -17.9±0.56 |
| 0.5 | -23.73±2.22 | -25.93±1.39 | -18.21±1.08 |
| 1 | -24.2±0.96 | -26.4±0.28 | -18.1±0.31 |
| 2 | -24.8±1.34 | -26.7±0.32 | -19.1±0.11 |
| 4 | -26.43±0.81 | -26.2±1.27 | -17.8±1.11 |
| 8 | -25.63±1.69 | -27.2±2.26 | -16.2±1.1 |
| 12 | -26.76±2.92 | -26.5±1.97 | -18.1±0.81 |
| 24 | -25.56±0.34 | -26.3±0.66 | -17.9±0.84 |


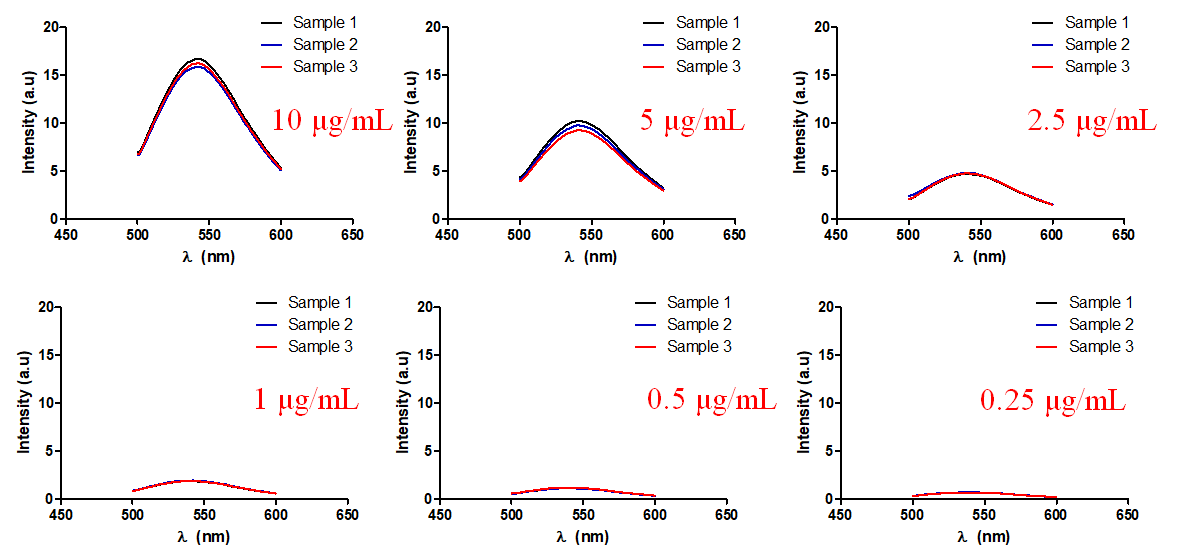


**Figure S1.** The raw data of fluorescence spectra for calibration curve.

**Figure S2.** The calibration curve for Cur quantification by using fluorescent method.


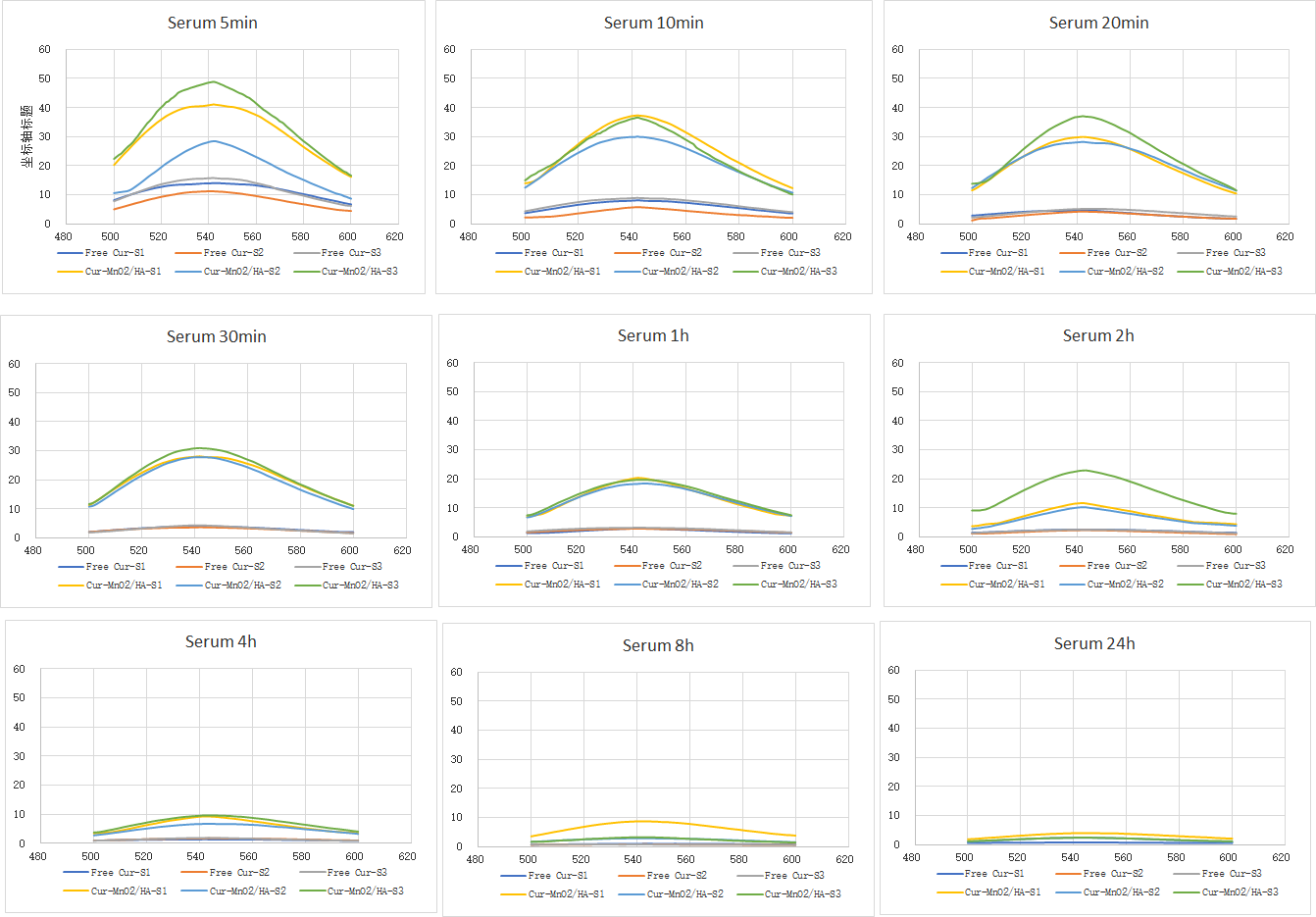


**Figure S3.** The raw data showing the fluorescent spectra of serum samples for both free Cur and Cur-MnO2/HA.


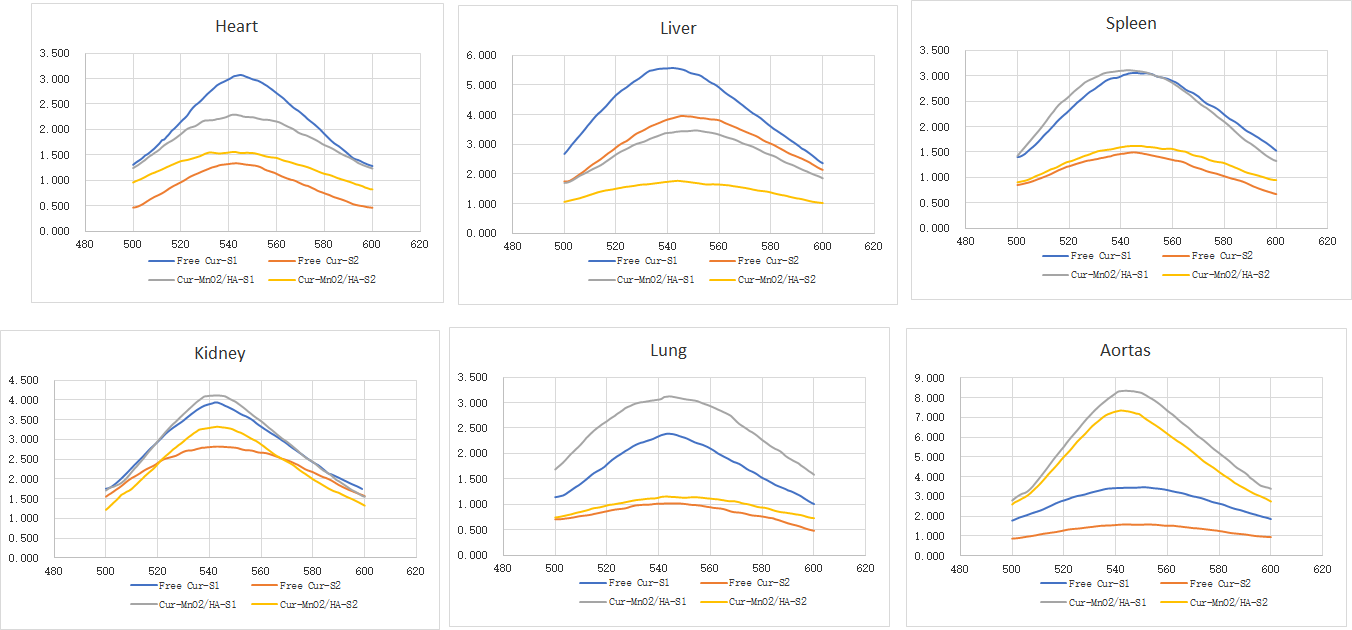


**Figure S4.** The raw data showing the fluorescent spectra of tissue samples for both free Cur and Cur-MnO2/HA.


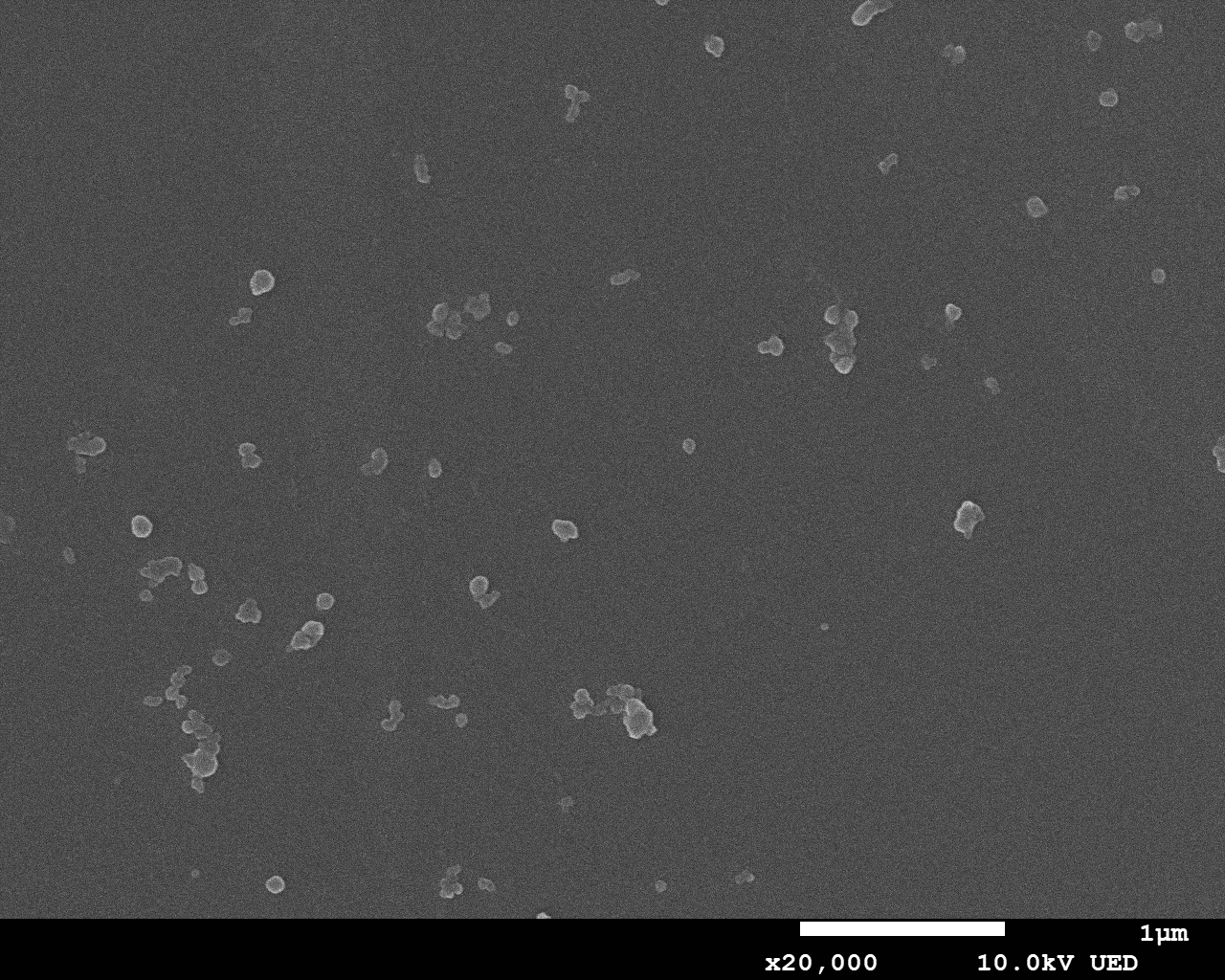


**Figure S5.** SEM micro-image of MnO_2_/HA.


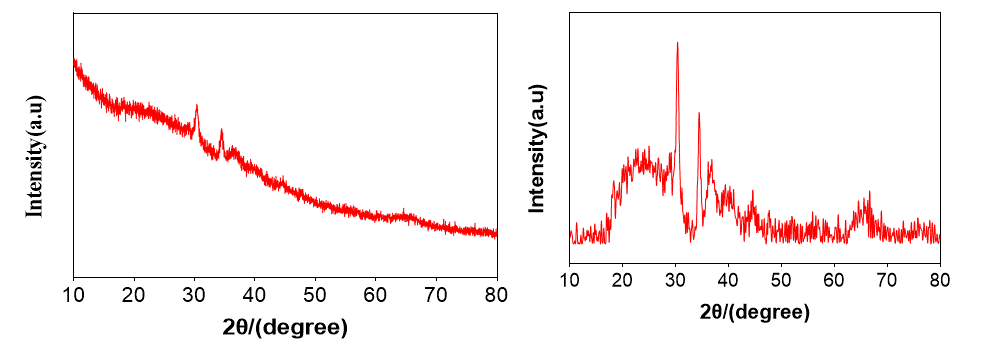


**Figure S6.** XRD spectra of MnO_2_/HA.

**Figure S7.** ζ potential of the nanoparticles before and after loading Cur.

**Figure S8.** Desorption of Cur from Cur-MnO_2_/HA by adding various probing ligands.

**Figure S9.** Dynamic colloidal stability of Cur-MnO_2_/HA NPs in water and different media by measuring particle size.


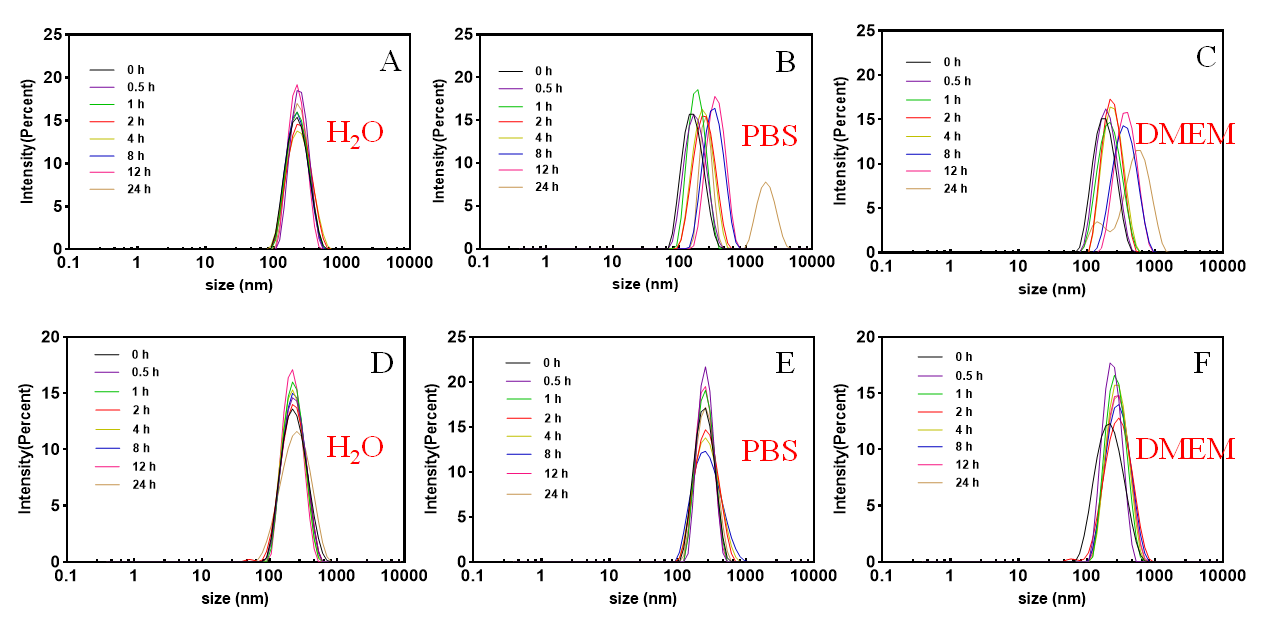


**Figure S10.** The representative raw data of dynamic size change for (A-C) MnO_2_/HA NPs and (D-F) Cur-MnO_2_/HA NPs under various media. It seems that the colloidal stability was enhanced after Cur loading, likely due to the increase of negative charge for stronger charge repulsion.


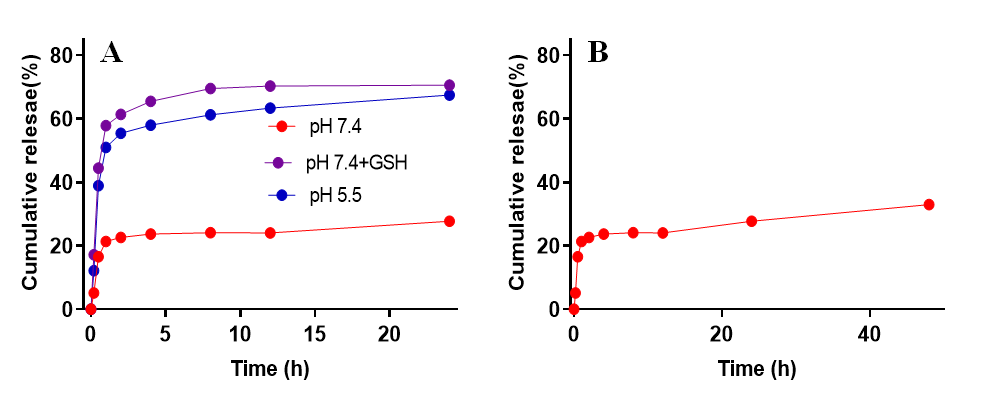


**Figure S11.** (A) Kinetics of drug release from Cur-MnO_2_/HA NPs in different dissolution media. (B) Kinetics of drug release from Cur-MnO_2_/HA NPs in PBS buffer (pH 7.4) up to 48 h.

**Figure S12.** Catalase-mimic activity of MnO_2_/HA in presence of 1 mM H_2_O_2_ by measuring the dynamic O_2_ generation.


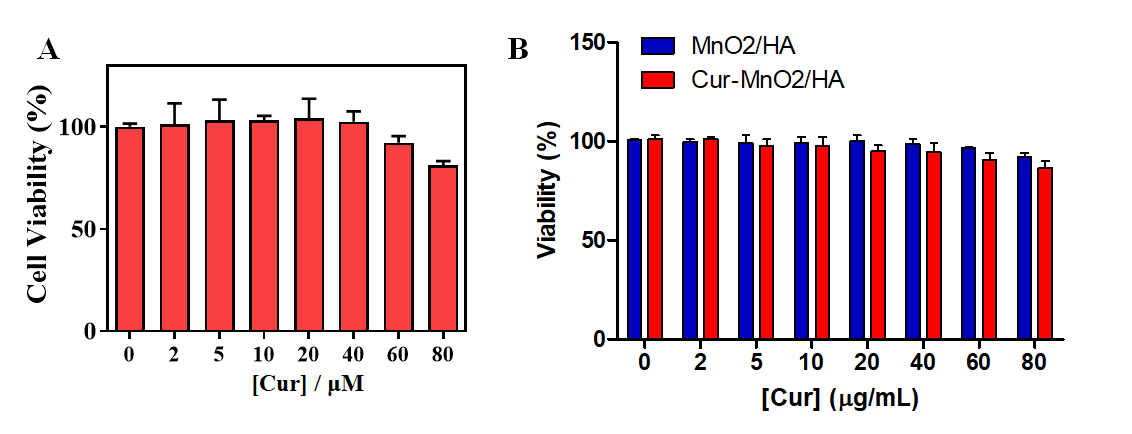


**Figure S13.** (A) Cell viability of the macrophages after incubating with MnO_2_/HA NPs at different equivalent Cur concentrations for 24 h. (B) Cell viability of the MOVAS cells after incubating with MnO_2_/HA NPs and Cur-MnO_2_/HA at different equivalent Cur concentrations for 24 h.


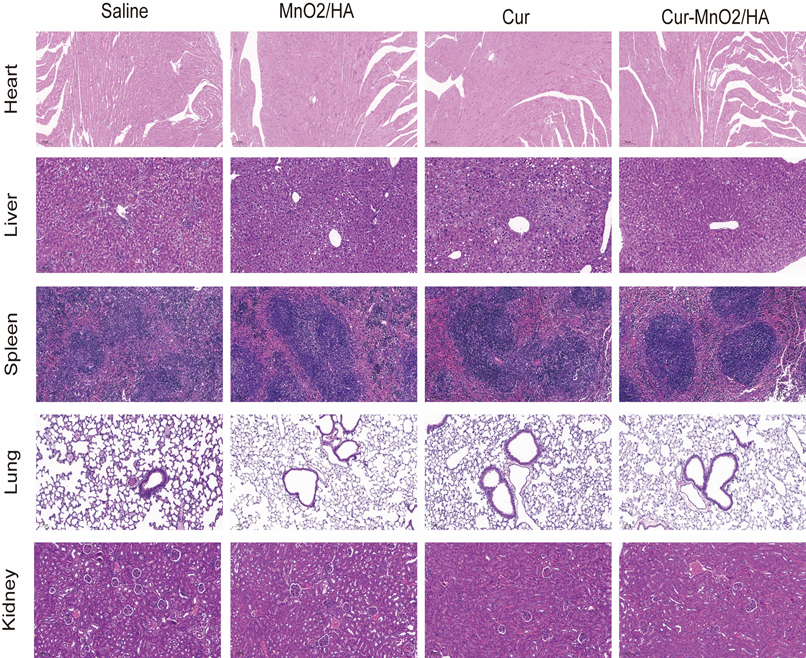


**Figure S14.** H&E staining of the major organs of mice after various treatments.
